# Comprehensive analysis of metabolism-related lncRNAs related to the progression and prognosis in osteosarcoma from TCGA

**DOI:** 10.1186/s13018-021-02647-4

**Published:** 2021-08-23

**Authors:** Xingyin Chen, Zhengyun Ye, Pan Lou, Wei Liu, Ying Liu

**Affiliations:** 1 Spinal Surgery, The First People’s Hospital of Jingmen, Jingmen, Hubei China; 2 Department of Gastroenterology, The First People’s Hospital of Jingmen, Xiangshan Avenue 168, Jingmen, 448000 Hubei China

**Keywords:** Osteosarcoma, Metabolism-related lncRNA, Prognosis signature, GSEA

## Abstract

**Background:**

Osteosarcoma is one of the most common malignant neoplasms in children and adolescents. Studies have shown that metabolism-related pathways are vital for the development and metastasis of osteosarcoma. Long non-coding RNA (lncRNA) plays a key role in the occurrence and progression of cancer in a variety of ways. However, the detailed molecular mechanisms of metabolism-related lncRNA in osteosarcoma remain to be deeply elucidated.

**Methods:**

In this study, all metabolism-related mRNAs and lncRNAs in osteosarcoma were extracted and identified based on transcriptomic data from the TCGA database. Usingsurvival analysis, univariate and multivariate independent prognostic analysis, gene set enrichment analysis, and nomogram, a prognostic signature with metabolic lncRNAs as prognostic factors was constructed.

**Results:**

Nine prognostic factors included lncRNA AC009779.2, lncRNA AL591895.1, lncRNA AC026271.3, lncRNA LPP-AS2, lncRNA LINC01857, lncRNA AP005264.1, lncRNA LINC02454, lncRNA AL133338.1, and lncRNA AC135178.5, respectively. Survival analysis indicated that alterations of specific lncRNA expression were strongly correlated with poor prognosis in osteosarcoma. Univariate and multivariate independent prognostic analysis showed that the prognostic signature had a good independent predictive ability for patient survival. The results of GSEA suggested that these predictors may be involved in the metabolism of certain substances or energy in cancer. The nomogram was further drawn for clinical guidance and assistance in clinical decision-making.

**Conclusions:**

This study identified multiple metabolism-related lncRNAs, which may be novel therapeutic targets for osteosarcoma, and contributed to better explore the specific metabolic regulatory mechanisms of lncRNA in osteosarcoma.

**Supplementary Information:**

The online version contains supplementary material available at 10.1186/s13018-021-02647-4.

## Introduction

Osteosarcoma, one of the most common Sarcoma, is usually diagnosed during childhood and adolescence [1]. Due to always occurring in the metaphysis of long bones and progressing rapidly, it often results in inconvenient or even restricted movement of patients [1]. Although effective progress has been made in current amputation and chemotherapy, patient prognosis is far from satisfactory, with 20% of patients metastasized at diagnosis [2]. Patients with metastatic osteosarcoma have a lower survival rate [3], which demands a better understanding of the mechanisms underlying osteosarcoma progression and metastasis. Therefore, it is particularly important to explore new therapeutic targets and accurate prognostic markers. Long non-coding RNA (lncRNA) has been shown to play an important regulatory role in the occurrence, development, and metastasis of all kinds of cancers [4–6]. Recently, the research on the abnormal expression of lncRNA in osteosarcoma has become ever more in-depth [7, 8]. On the other hand, growth of bone tissue is inextricably linked to bone metabolism [9]. Abnormal bone metabolism can lead to loss of control of bone growth or abnormal differentiation, which further results in osteoma or osteosarcoma [9]. Despite many therapeutic targets and prognostic markers for osteosarcoma have been reported, such as immunomodulation, tumor infiltrating immune cells could be used as a prognosis factor in osteosarcoma patients [10], *Niu et. al* had found that EGR1, CXCL10, MYC and CXCR4 could be regarded as potential biomarker of osteosarcoma [11]. Few reports show that lncRNA affects osteosarcoma by regulating the metabolic process [12], and the roles of most lncRNAs in regulating the metabolism of osteosarcoma remain unclear.

In this study, gene expression data and clinical information were available from The Cancer Genome Atlas (TCGA) database. Metabolic genes and related lncRNAs were further extracted. Nine Metabolism-related lncRNAs (MRlncRNAs) associated with high prognosis were finally obtained with various bioinformatics analysis methods. We further constructed and verified a prognostic signature of osteosarcoma. This study explains the possible metabolic epigenetic mechanism and provides new therapeutic targets of osteosarcoma.

## Materials and methods

### Data downloading and processing

All transcriptions data of osteosarcoma samples were downloaded from the TCGA database [13]. There was a total of 265 samples. After removing duplicated and extremely low expression genes, the mRNA and lncRNA matrixes were extracted, respectively. The c2.cp.kegg.v7.2.Symbols.Gmt from the Gene Set Enrichment Analysis (GSEA) database was downloaded [14], a total of 940 related genes in metabolic pathways were extracted. Furthermore, the intersection of the extracted mRNA matrix was taken to obtain the expression data of 935 metabolic genes.

### Preliminary Screening of MRlncRNA

By using the correlation test in R 4.0.3, 335 lncRNAs that highly related to metabolic genes (corFilter = 0.6, pvalueFilter = 0.001) were acquired. 15 lncRNAs significantly related to prognosis were obtained by combining with year of survival through Kaplan–Meier survival (*P* < 0.05) analysis and univariable Cox regression survival (*P* < 0.05) analysis.

### Construction and evaluation of prognostic signature

We used “glmnet” package in R 4.0.3 to perform Least Absolute Shrinkage and Selection Operator (LASSO) regression analysis, and further used the multivariable Cox regression analysis to obtain prognosis-related metabolic lncRNA. These factors were used to construct a prognostic signature. Then, the risk score signature for prognostic metabolism of lncRNA was established according to the following formula: Risk Score (RS) = *∑β*_*(MRlncRNA)*_ × *Exp*_*(MRlncRNA)*_. *β* represents the coefficient of the multivariable Cox regression analysis. *Exp*_*(MRlncRNA)*_ means the individual prognostic metabolic lncRNA expression value. The risk score of each osteosarcoma patient was calculated accurately, then, all patients were divided into high-risk and low-risk groups through the median risk score. In addition, a survival curve was drawn to assess the difference in survival between the two groups by utilizing the “survival” package in R 4.0.3.

### Metabolism-related mRNA-lncRNA network construction

Nine hundred thirty five metabolic genes and 335 MRlncRNAs extracted from the TCGA database were utilized to construct the co-expression network. Similarly, we also constructed the network of lncRNAs and related metabolic genes with prognostic signatures. The visual co-expression network was produced by Cytoscape 3.7.2.

### Independent prognosis and clinical correlation analysis

Univariate and multivariate Cox regression analyses were applied to determine independent clinical prognostic factors, that is, whether individual lncRNA has a potential impact on certain clinical features or not. *P* value < 0.05 was considered statistically significant.

### Survival analysis

By using the R “survival” package, the risk model was analyzed and evaluated whether the high- or low-risk group was related to survival. Similarly, each prognostic factor was divided into high- and low-expression groups according to the median expression value, then the survival curve was drawn. *P* value < 0.05 was statistically significant.

### Nomogram drawing and evaluation

The nomogram was drawn with the known clinicopathological data and the risk score. Firstly, the receiver operator characteristic (ROC) curve was used to evaluate its representativeness. Secondly, the C index was used to evaluate its predictive ability. In addition, the calibration curves of 1-year, 3-year and 5-year survival rates were established to evaluate the accuracy of the nomogram. Finally, to reduce the dimensionality of the data and figure out whether or not an individual lncRNA could distinguish patients' risk groups, we performed principal component analysis (PCA).

## Result

**Fig. 1:**
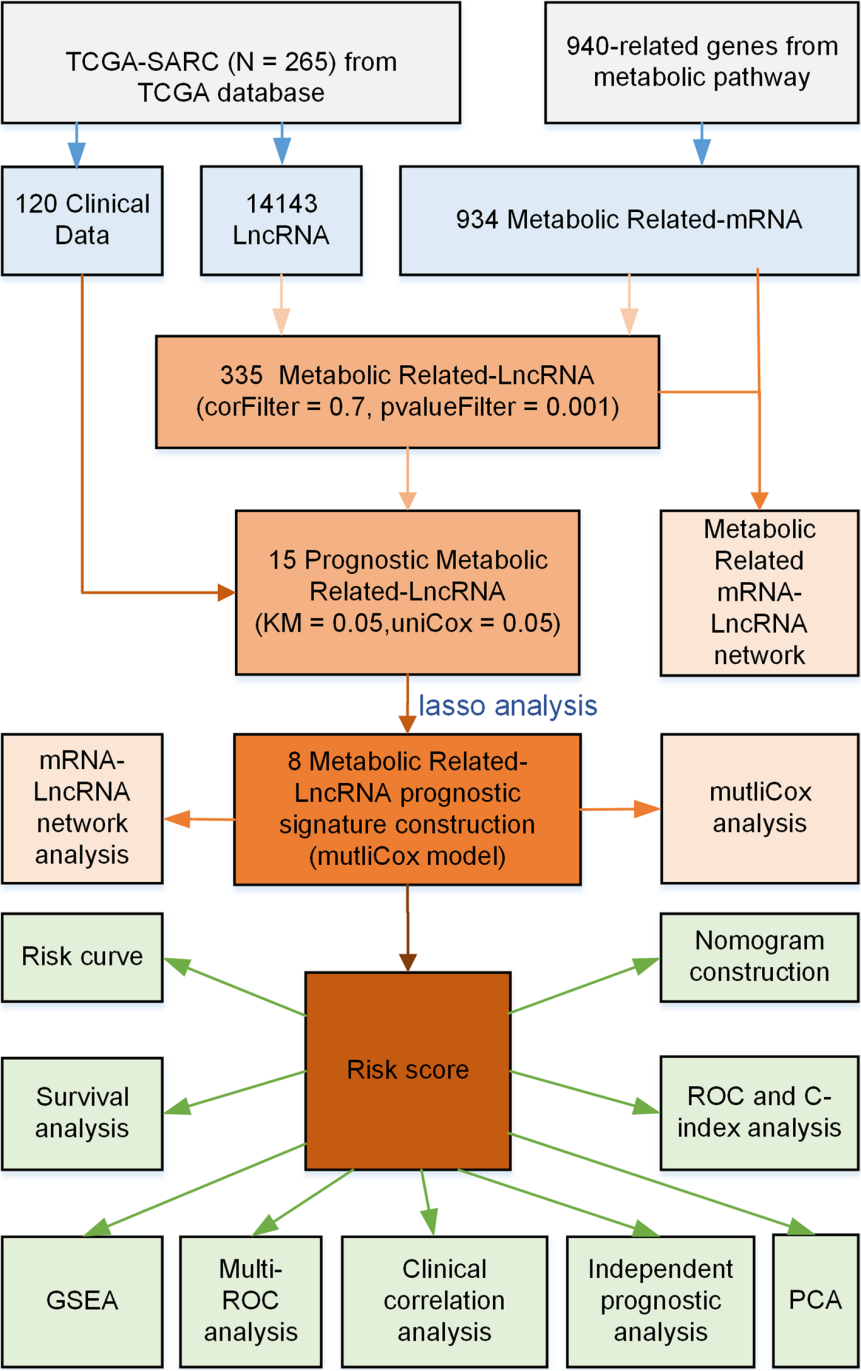
Flow diagram of entire data analysis

The entire research process is shown in Fig. 1.

### Screening of MRlncRNAs

**Fig. 2:**
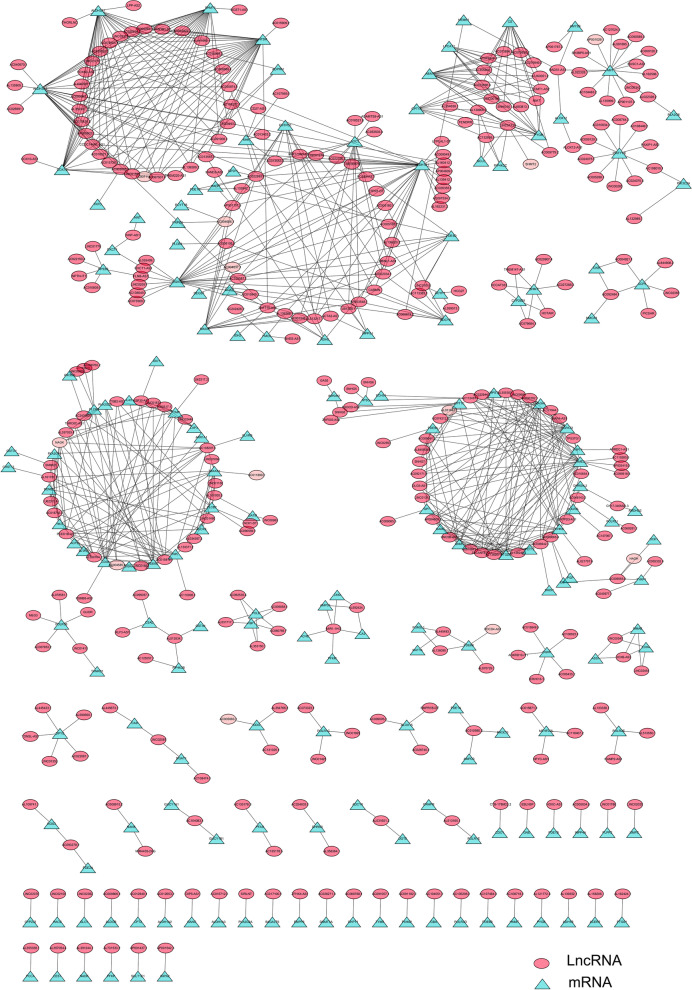
mRNA-lncRNA network of 935 metabolic genes and 335 metabolic-related lncRNAs

By downloading the transcriptions data of osteosarcoma patients, the mRNA and lncRNA matrixes were extracted, respectively. A total of 940 genes related to metabolic pathways were acquired from the GSEA database. The expression data of 935 metabolic genes were extracted accurately. And 335 lncRNAs, which are closely correlated with metabolic genes, were obtained by correlation test in the R. These lncRNAs. A network of 935 metabolic genes and 335 related lncRNA was constructed and visualized by Cytoscape (Fig. 2). Most mRNAs and lncRNAs in this network have a one-to-many or many-to-one relationship, which initially reveals the complexity and extensiveness of lncRNAs network regulation. After incorporating the complete prognostic information, we utilized Kaplan–Meier survival analysis and univariate Cox regression survival analysis to obtain 15 lncRNAs significantly related to the prognosis (Supplementary Table 1).

### Construction and evaluation of risk signature

**Fig. 3:**
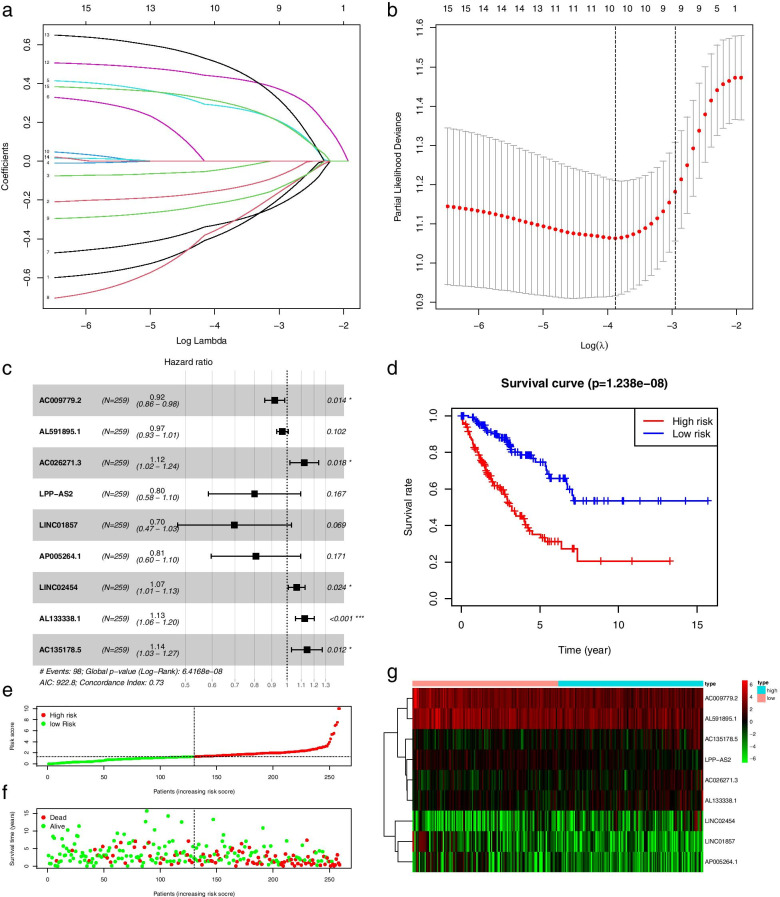
The prognostic signature was established and validated based on nine prognostic metabolic lncRNAs. **a** LASSO coefficient profiles of the lncRNAs associated with the metabolism of osteosarcoma, **b** Partial likelihood deviance is plotted versus log (λ). **c** The forest map shows multivariable COX analysis of prognostic signatures. **d** The survival curve indicates the difference between high and low risk group. **e** The risk curves were reordered based on the risk score of each patient. **f** The scatter plot displayed the overall survival in osteosarcoma patients. **g** The heatmap revealed the expression levels of metabolic lncRNAs

To further reduce and optimize the signature, the R “glmnet” package was used to perform LASSO regression analysis, and the model showed best fits when λ = -3 and N = 9 (Fig. 3a-b). We further used the multivariate Cox regression analysis to obtain nine prognostic metabolic lncRNA and drew a forest map (Fig. 3c). It reveals that lncRNA AC026271.3, lncRNA LINC02454, lncRNA AL133338.1, and lncRNA AC135178.5 are high-risk factors for patient survival time and status, which implies that these four lncRNAs may act as carcinogens. Since lncRNA AC009779.2 is a low-risk factor, it is very likely to become a protective factor. Then we used these 9 factors to construct a prognostic risk signature:

Risk Score (RS) = AC009779.2*-0.08566 + AL591895.1*-0.03249 + AC026271.3*0.116683 + LPP-AS2*-0.22239 + LINC01857*-0.35896 + AP005264.1*-0.21311 + LINC02454*0.065331 + AL133338 0.1*0.120016 + AC135178.5*0.134252.

According to this formula, regarding the median risk score as the grouping standard, we can accurately calculate the risk score of each osteosarcoma patient. Then all patients were subdivided into high- or low-risk groups. The survival difference between the two groups was evaluated by drawing a survival curve (Fig. 3d). Patients in the low-risk group were under a higher survival rate while patients in the high risk group had a worse survival rate (*P* < 0.001). In addition, we drew a risk curve and a heat map of the expression of the nine lncRNAs in all samples based on the constructed signatures (Fig. 3e-g). This signature can accurately distinguish high-risk patients from low-risk groups through different risk score and survival time.

### Network construction of lncRNAs and related mRNAs

**Fig. 4:**
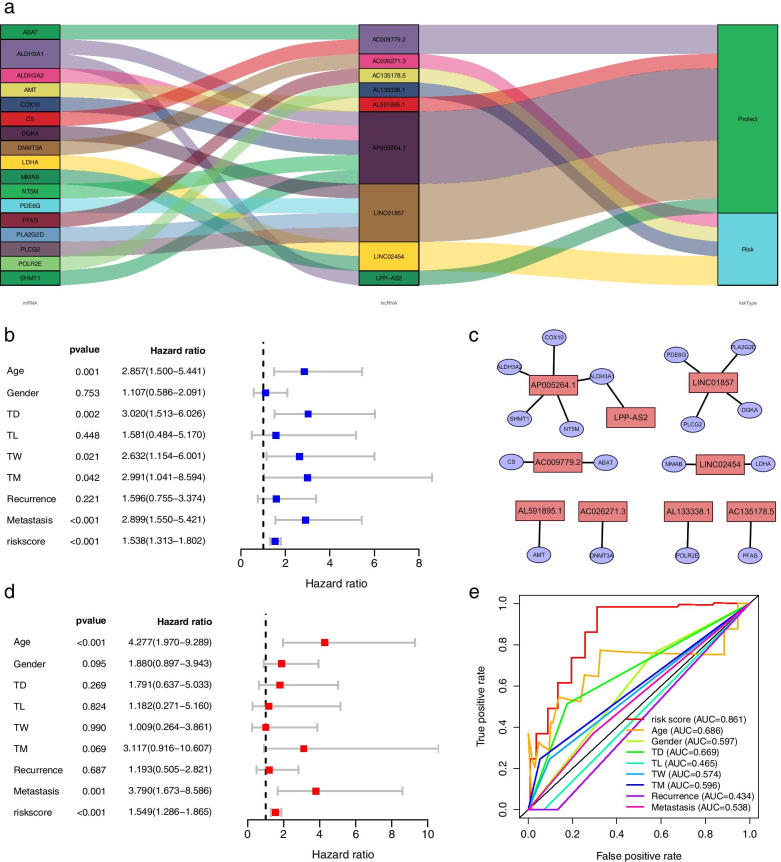
mRNA-lncRNA network of nine prognosis-related metabolic lncRNA and independent prognostic analysis. **a** The Sankey diagram showed nine metabolic lncRNAs in the mRNA-lncRNA network. **b** The univariate Cox analysis of independent prognostic signature. **c** Nine metabolic lncRNAs in the mRNA-lncRNA network in Cytoscape. **d** The multivariate Cox analysis of independent prognostic signature. **e** The multi-index ROC curves among prognostic signature and other clinical features

In the next step, we also constructed a grid of nine lncRNAs and their related metabolic genes. Figure 4a shows a Sankey diagram constructed based on co-expression relationships. The left part shows related metabolic genes. The middle part indicates lncRNAs that are highly related to metabolic genes, and the right part represents prognostic types of lncRNAs. For example, LINC01857 may have a co-expression relationship with DGKA, PLCG2, and PLA2G2D, which means that some interactions or mutual regulation mechanisms exist between them. In addition, lncRNA LINC01857 shows a protective role, suggesting that it may act as a tumor suppressor gene in the development of cancer. Similarly, results of Cytoscape 3.7.2 (Fig. 4c) intuitively reveal the mRNA-lncRNA co-expression relationships.

### Independent prognosis and clinical correlation analysis

**Fig. 5:**
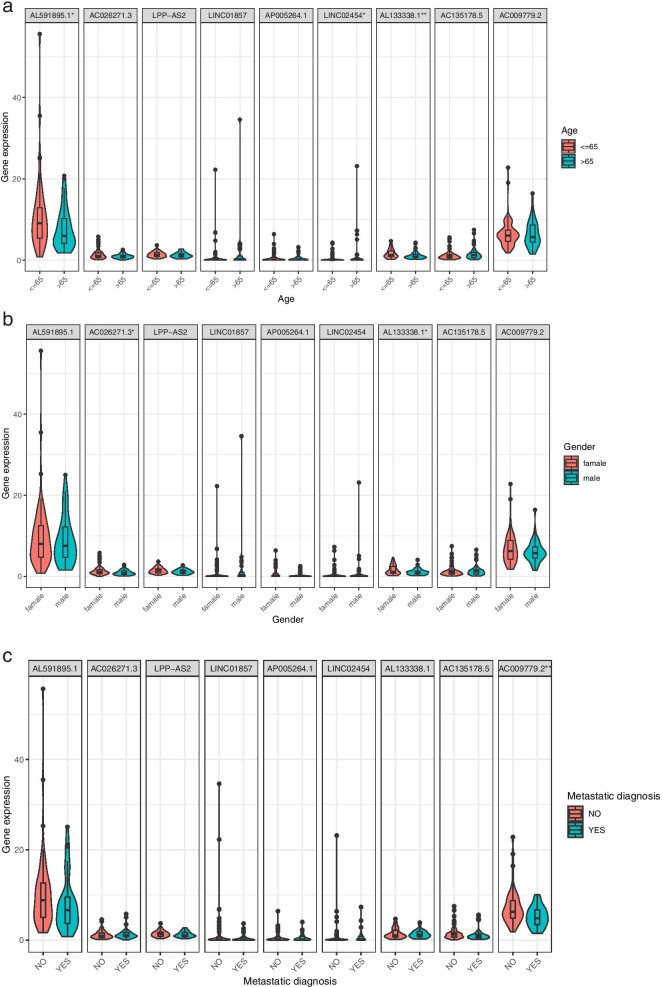
Correlation analysis of clinical features and individual metabolic lncRNA. Violin diagrams of correlations between (**a**) age, (**b**) gender, and (**c**) metastasis and the expression value of each metabolic lncRNA

Next, to determine whether it applies to guide clinical decision-making or assist in evaluating the prognosis, we verified the independent prognostic capacity of the prognostic signature constructed above by using the univariate Cox regression analysis respectively. In the univariate Cox regression analysis, the hazard ratio of the risk score is 1.538 (1.313–1.802) and is statistically significant (*P* < 0.001) (Fig. 4b). Similarly, in the multivariate regression analysis, the hazard ratio of the risk score is 1.549 (1.286–1.865) and reveals a significant statistical significance (*P* < 0.001) (Fig. 4d). Furthermore, the multi-index ROC curve showed that the risk score has better diagnostic and recognition capabilities for patients with osteosarcoma (AUC = 0.861) than other clinical features (Fig. 4e). In addition, we further analyzed whether a single lncRNA has a potential impact on certain clinical characteristics (Fig. 5). For example, as shown in Fig. 5a, lncRNA AL591895, lncRNA LINC02454, and lncRNA AL133338.1 show definite differences in tumor populations over and under 65 years of age and younger (*P* value < 0.05). Similarly, lncRNA AC026271.3 and lncRNA AL133338.1 also show partial expression differences for patients of different genders (*P* value < 0.05) in Fig. 5b. LncRNA AC009779.2 appears to be related to cancer metastasis (*P* value < 0.01) in Fig. 5c.

### Prognostic survival analysis of MRlncRNA

**Fig. 6:**
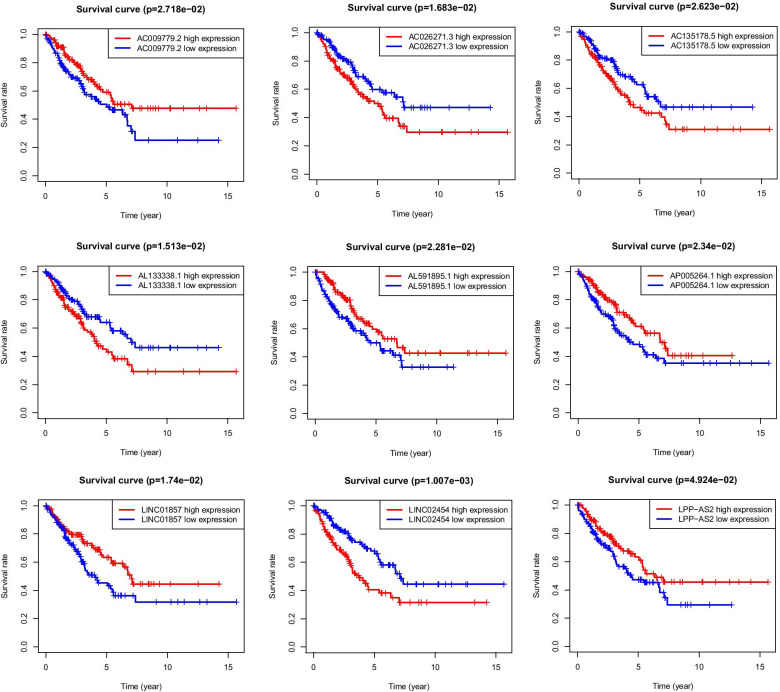
Survival analysis. The survival curve displayed the different overall survival between high- and low-expression groups of each metabolic lncRNA

To further evaluate the independent prognostic capacity of single lncRNA in the signature, we divided each lncRNA into high- and low-expression groups according to the median expression value and drew the survival curve (Fig. 6). It reveals that all nine prognostic factors are statistically significant, and patients with two different prognoses can be distinguished. It further illustrates that they may play potential anti-cancer or carcinogenic roles in osteosarcoma.

### GSEA analysis

**Fig. 7:**
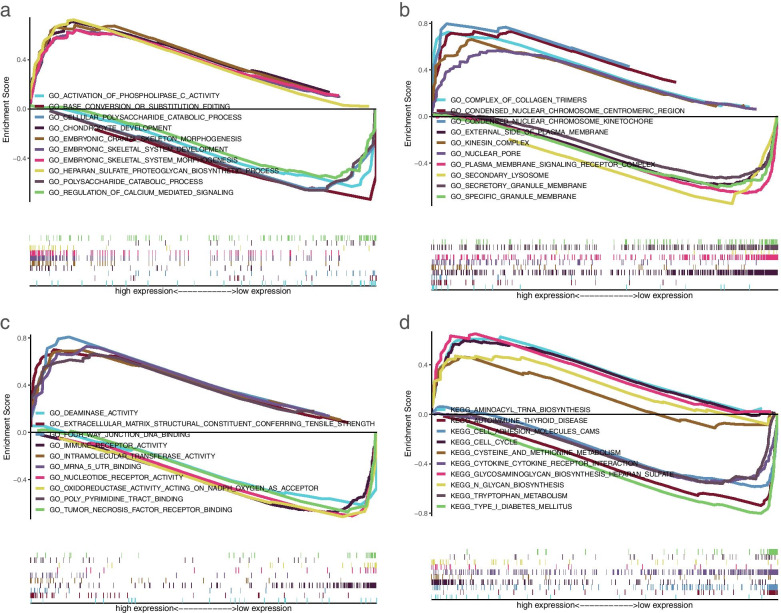
GSEA analysis. A multi-GSEA plot of the main enrichment pathways of BP (**a**), CC (**b**), and MF (**c**) in GO. A multi-GSEA plot of the main enrichment pathways of KEGG (**d**)

Signed risk scores were divided into high- and low-risk groups according to the median value, and GSEA analysis was performed on all samples (Fig. 7). In the biological process of gene ontology (GO), we can see the enrichment of multiple biological processes related to bone metabolism. The high-risk groups are significantly enriched in CHONDROCYTE DEVELOPMENT, EMBRYONIC CRANIAL SKELETON MORPHOGENESIS, EMBRYONIC SKELETAL SYSTEM DEVELOPMENT, EMBRYONIC SKELETAL SYSTEM MORPHOGENESIS and HEPARAN SULFATE PROTEOGLYCAN BIOSYNTHETIC PROCESS. The low-risk groups are significantly enriched in the ACTIVATION OF PHOSPHOLIPASE C ACTIVITY, BASE CONVERSION OR SUBSTITUTION EDITING, POLYSACCHARIDE CATABOLIC PROCESS, POLYSACCHARIDE CATABOLIC PROCESS and REGULATION OF CALCIUM MEDIATED SIGNALING pathways (Fig. 7a). Figure 7b and c reflect the cell component and molecular function of the high- and low-risk groups, respectively. Figure 7d shows the aggregation of metabolic pathways and cancer pathways in the high- and low-risk groups in KEGG. In conclusion, GSEA analysis further describes the potential biological functions of prognostic factors.

### Nomogram drawing and PCA

**Fig. 8:**
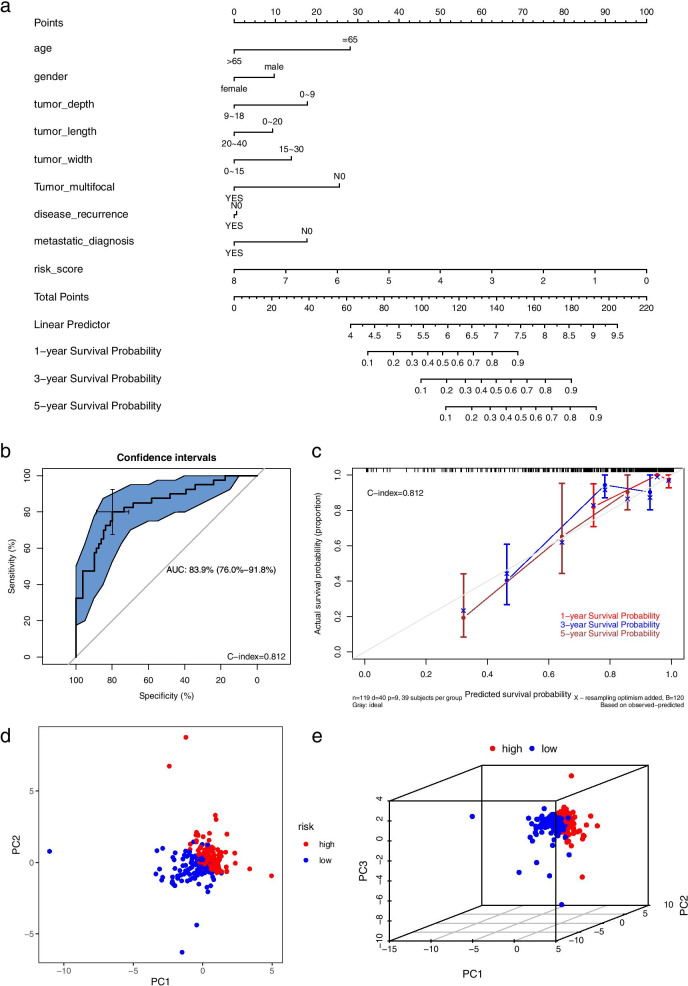
Drawing the nomogram and principal component analysis. **a** The nomogram consisted of the risk score and multiple clinical features. **b** The ROC curve in the nomogram. **c** The correction curve in the nomogram. **d** PCA 2d and (**e**) PCA 3d

To make the signature to facilitate clinical prognosis guidance, we drew a nomogram by using available clinical-pathological data and risk score we signed (Fig. 8a). Then, the ROC curve (AUC = 0.839) indicates the nomogram is well representative (Fig. 8b). Further the C index (0.812) establishes that the nomogram has a good distinguishing performance. In addition, we also developed a calibration curve using 1-year, 3-year, and 5-year survival rates to estimate the accuracy of the nomogram (Fig. 8c). It indicates that the nomogram evaluation of the 3-year survival rate is the most accurate. Finally, to better reflect the difference between the high- and low-risk of each prognostic lncRNA, we conducted a principal component analysis of these prognostic factors. 2d-PCA (Fig. 8d) and 3d-PCA (Fig. 8e) analysis both show that the prognostic signature composed of these lncRNAs can distinguish precisely whether the patient belongs to the low- or high-risk group after dimensionality reduction of the data. Itonce again verifies the accuracy of the signature.

## Discussion

Sarcoma is a malignant tumor derived from connective tissue or other non-epithelial tissues [15]. It occurs in a variety of tissues and organs, including osteosarcoma, leiomyoma, lymphosarcoma, and synovial sarcoma [16], among which osteosarcoma is most common in children and adolescents [17]. Due to the active cartilage and bone metabolism in children and adolescents, osteosarcoma develops rapidly and makes early metastasis [18]. Therefore, the standard of treatment is amputation or chemotherapy [18]. However, 20% of patients are prone to early metastasis at diagnosis [2], so it is necessary to explore the pathogenesis and metastasis mechanism. Tumor cells are extremely active in their internal energy and material metabolism due to their immortal proliferation [9]. Several studies have reported the molecular mechanisms about the specific metabolic process of osteosarcoma cells in the body, which proves the key role of the regulation of metabolic pathway in the process of osteosarcoma [9, 19, 20].

In recent years, with the rapid development of high-throughput technology, the mechanism of various diseases including cancer has been studied more and more deeply, especially in epigenetics, which is represented by lncRNA and circRNA [4, 5]. Sequencing technology identified five lncRNA types including sense, antisense, bidirectional, intergenic and intronic lncRNAs [21, 22]. These 5 lncRNAs work in different ways. Despite increasing lncRNAs have been reported in osteosarcoma, comparatively few reports reveal roles of lncRNAs in the metabolism of osteosarcoma. Our present study firstly screened out MRlncRNAs in osteosarcoma through comprehensive bioinformatics analysis. It provides guidance for further researches about the molecular mechanism of osteosarcoma metabolism.

In this study, we obtained nine metabolism-related lncRNAs in osteosarcoma, including lncRNA AC009779.2, lncRNA AL591895.1, lncRNA AC026271.3, lncRNA LPP-AS2, lncRNA LINC01857, lncRNA AP005264.1, lncRNA LINC02454, lncRNA AL133338.1 and lncRNA AC135178.5. In addition, we constructed a prognostic signature and the risk score based on the signature. Survival analysis indicates substantially different prognoses between the high- and low-risk groups. The multi-index ROC curve shows that the diagnostic power of the prognostic signature is preferable to clinical factors. At the same time, we also found some lncRNAs were closely related to clinical features such as age, sex and tumor metastasis. All prognostic factors can independently distinguish the prognostic survival rate of patients. GSEA analysis shows that the high- and low-risk groups are closely linked to cancer and multiple metabolic pathways. Finally, we constructed a nomogram to quantify clinical prognosis.

Some of these nine prognostic factors have been reported in other cancers, such as LPP-AS2. LPP-AS2 is thought to be associated with immune cells in soft tissue sarcoma [23]. Another study reported that LPP-AS2 regulates EGFR expression by sponging Mir-7-5p as a ceRNA and combination of the promoter region of LPP-AS2 with c-MYC contributes to the development of glioma [24]. LINC01857 has been reported to predict prognosis in hepatocellular carcinoma patients with fibrosis [25]. Another study showed its potential as a novel molecular marker for pancreatic cancer [26]. LINC01857 also can bind to promoters to regulate the transcription process of protein. For example, by interacting with CREBBP to promote the transcription of H3K27Ac and CREB1, LINC01857 promote the progression of breast cancer [27]. In addition, another function of LINC01857 is to combine with miRNAs to play a spongy effect. For example, LINC01857 sponges miR-1281 to promote TRIM65 expression and thus facilitates the development of glioma [28]. A recent study revealed that LINC01857 promotes the migration and invasion of gastric cancer cells by regulating microRNA-200b [29]. LINC02454, as a long intergenic lncRNA, is mainly reported in papillary thyroid carcinoma [30, 31]. In addition, other lncRNAs in this study have not been reported in the relevant literature. It suggests that the prognostic signature we constructed is worth in-depth study. Although we integrated a variety of bioinformatics methods and statistical methods, and used various analyses to construct prognostic signatures and multiple verifications, several limitations remain. First, the samples come from a single database, which may be lack of representativeness. Second, no in vivo or in vitro studies were performed. Both the above deficiencies are also the main aspects of our further research.

## Conclusion

We constructed a prognostic signature composed of lncRNAs for the metabolic process of osteosarcoma, which may provide guidance for clinical activities and assist clinical decision-making. In addition, it provided a reference for further study on the regulation of lncRNA on osteosarcoma metabolism.

## Supplementary Information

**Additional file 1:Supplementary Table 1**. Fifteen Metabolism-related LncRNA were acquired precisely by Kaplan-Meier survival (*P* < 0.05) analysis and univariable Cox regression survival (*P* < 0.05) analysis.

## Data Availability

All data generated or analyzed during this study are included in this published article and its Additional files.

## Abbreviations

MRlncRNA: Metabolism-related lncRNA
LASSO: Least absolute shrinkage and selection operator
PCA: Principal component analysis
ROC: Receiver operator characteristic
TD: Tumor depth
TL: Tumor length
TW: Tumor width
TM: Tumor multifocal
CREB1: Cyclic AMP-responsive element-binding protein 1
CREBBP: CREB-binding protein
