# Supplementary material for: Comprehensive analysis of metabolism-related lncRNAs related to the progression and prognosis in osteosarcoma from TCGA
Source: J Orthop Surg Res. 2021 Aug 23;16:523. doi: 10.1186/s13018-021-02647-4 (PMC8381543; doi:10.1186/s13018-021-02647-4)
Supplement: Supplementary file 1 — Additional file 1:Supplementary Table 1. Fifteen Metabolism-related LncRNA were acquired precisely by Kaplan-Meier survival (P < 0.05) analysis and univariable Cox regression survival (P < 0.05) analysis. [file 13018_2021_2647_MOESM1_ESM.docx]

**Comprehensive analysis of Metabolism-related lncRNAs** **related to the progression and prognosis in osteosarcoma from TCGA**

Xingyin Chen ^1^, Zhengyun Ye^1^, Pan Lou^1^, Wei Liu^1^, Ying Liu ^2,^ *****

^1^Spinal surgery, The First People’s Hospital of Jingmen, Jingmen, Hubei, China.

^2^Department of Gastroenterology, The First People’s Hospital of Jingmen, Jingmen, Hubei, China.

***Corresponding Author**: Dr. Ying Liu, The First People’s Hospital of Jingmen, Xiangshan Avenue 168, Jingmen 448000, Hubei, China. E-mail: [rainly.1988@163.com](mailto:rainly.1988@163.com)

| gene | KM | HR | HR.95L | HR.95H | P value |
| --- | --- | --- | --- | --- | --- |
| AC009779.2 | 0.027177761 | 0.917458595 | 0.860239001 | 0.978484202 | 0.008742656 |
| AL591895.1 | 0.022811453 | 0.95913997 | 0.925648733 | 0.993842965 | 0.021417565 |
| AC055874.1 | 0.010962534 | 0.74843908 | 0.592110537 | 0.946041359 | 0.015351468 |
| ZFHX4-AS1 | 0.015397412 | 1.021764832 | 1.001423104 | 1.042519758 | 0.035854894 |
| AC026271.3 | 0.016829963 | 1.160904344 | 1.053806105 | 1.27888697 | 0.002517682 |
| AL357033.4 | 0.017765972 | 0.654180637 | 0.481770326 | 0.888291128 | 0.00655005 |
| LPP-AS2 | 0.04923931 | 0.705594932 | 0.518473407 | 0.960250231 | 0.026558293 |
| LINC01857 | 0.017399306 | 0.610565811 | 0.411528299 | 0.905868711 | 0.01424138 |
| AP005264.1 | 0.023400816 | 0.716708969 | 0.539461159 | 0.952194125 | 0.021566644 |
| AL034397.3 | 0.012633432 | 0.681315195 | 0.483844785 | 0.959378729 | 0.027989099 |
| PSMB8-AS1 | 0.016111038 | 0.941939078 | 0.902491558 | 0.983110832 | 0.006137823 |
| LINC02454 | 0.001006834 | 1.082175709 | 1.025574189 | 1.14190107 | 0.003960561 |
| AL133338.1 | 0.015130158 | 1.123847196 | 1.051906939 | 1.200707471 | 0.000541657 |
| AC107959.1 | 0.021548471 | 0.632130896 | 0.438792241 | 0.910657555 | 0.01380051 |
| AC135178.5 | 0.026227745 | 1.141057095 | 1.038542563 | 1.253690837 | 0.006007775 |

**Supplementary Table 1**

15 Metabolism-related LncRNA were acquired precisely by Kaplan-Meier survival (P < 0.05) analysis and univariable Cox regression survival (P < 0.05) analysis.
